# BST1 rs4698412 allelic variant increases the risk of gait or balance deficits in patients with Parkinson’s disease

**DOI:** 10.1111/cns.13099

**Published:** 2019-01-24

**Authors:** Yu‐Ting Shen, Jian‐Wei Wang, Min Wang, Yan Zhi, Jun‐Yi Li, Yong‐Sheng Yuan, Xi‐Xi Wang, Hui Zhang, Ke‐Zhong Zhang

**Affiliations:** ^1^ Department of Neurology The First Affiliated Hospital of Nanjing Medical University Nanjing China; ^2^ Department of Radiology The First Affiliated Hospital of Nanjing Medical University Nanjing China

**Keywords:** amplitude of low‐frequency fluctuations, BST1 rs4698412, imaging genetics, lingual gyrus, Parkinson’s disease

## Abstract

**Aims:**

We aimed to explore effects of bone marrow stromal cell antigen‐1 (BST1) rs4698412 allelic variant on brain activation and associative clinical symptoms in Parkinson’s disease (PD).

**Methods:**

A total of 49 PD patients and 47 healthy control (HC) subjects were recruited for clinical evaluations, blood samples collection for genotypes, and resting‐state functional MRI (rs‐fMRI) scans. Based on BST1 rs4698412 allelic variant (G → A), participants were further divided into 18 PD‐GG, 31 PD‐GA/AA, 20 HC‐GG, and 27 HC‐GA/AA carriers, which respectively indicated subjects carrying ancestral or risk allele in that locus in PD or HC. Two‐way analysis of covariance (ANCOVA) was applied to investigate main effects and interactions between PD and BST1 rs4698412 allelic variant on brain function via amplitude of low‐frequency fluctuations (ALFF). Spearman’s correlations were then utilized to detect associations between interactive brain regions and clinical symptoms.

**Results:**

Compared to HC subjects, PD patients exhibited increased ALFF values in left cerebellum_8 and cerebellum_9. Significant interaction was in right lingual gyrus, where there were the lowest ALFF values and ALFF values were only negatively associated with Timed Up and Go (TUG) test time in PD‐GA/AA subgroup.

**Conclusion:**

BST1 rs4698412‐modulated lingual gyrus functional alterations could be related to gait and balance dysfunction in PD.

## 1 INTRODUCTION

Parkinson’s disease (PD), a progressive neurodegenerative disorder, is attributed to degeneration of dopamine (DA) neurons in the nigrostriatal pathway and the underlying pathological mechanism is still unknown.^1^, ^2^ Mitochondrial dysfunction, oxidative stress, and inflammation were implicated in PD pathogenesis potentially,^3^, ^4^, ^5^ and in sporadic PD these processes are induced by nongenetic factors including environmental risk factors, probably in interaction with susceptibility genes.^4^, ^6^ Associated genes underlying susceptibility to PD have received increasing attention recently. Apart from the known PD genes (for example SNCA, LRRK2, MAPT), a new locus 4p15/bone marrow stromal cell antigen‐1 (BST1) was identified to participate in the PD progression.^7^ BST1, also known as CD157, plays a role in immune responses.^8^ In addition, BST1 could produce cyclic ADP‐ribose involved in regulating calcium homeostasis.^9^, ^10^ Either abnormal immune responses^5^ or imbalance of calcium homeostasis^11^, ^12^ could cause the development of PD. Moreover, accumulating evidence demonstrated the most investigated single nucleotide polymorphism (SNP) in BST1 gene was rs4698412 variant (G → A). A meta‐analysis 13 and genomewide association studies (GWAS)^7^, ^14^ in different populations have affirmed that BST1 rs4698412 was relevant to the increased risk of sporadic PD. Particularly, a study in Chinese population also manifested the association between BST1 rs4698412 and PD.^15^ Taken together, these results prompted the potential role of BST1 rs4698412 allele in the development of PD. However, these studies indicated little or no information regarding the association between BST1 rs4698412 allelic variant and clinical features, especially about how to mediate neural function or brain deficits in the pathogenesis of PD. Thus, in the current study, we aimed to explore the probable impact of BST1 rs4698412 allelic variant on brain functions and clinical symptoms in PD patients.

Imaging genetics could provide a more thorough and precise insight into the influence of gene variants on the brain.^16^ Meanwhile, imaging genetics has been applied in research of multiple diseases including Alzheimer’s disease (AD)^17^ and attention‐deficit/hyperactivity disorder.^18^ Resting‐state functional magnetic resonance imaging (rs‐fMRI) can indicate the phenomenon of spontaneous neuronal activity at rest by examining spontaneous fluctuations in the blood oxygen level dependent (BOLD) signal of brain regions without any explicit stimulation.^19^Therefore, it could provide a platform to explore the probable gene function in the brain. Amplitude of low‐frequency fluctuations (ALFF) reflects the level of regional brain activation by detecting the spontaneous amplitude of low‐frequency (0.01‐0.08 Hz) BOLD signal.^20^ A series of studies have applied ALFF approach to focus on possible pathogenesis in PD previously. For example, a study revealed that ALFF analysis could sensitively and specifically distinguish individuals with PD in the off medication state from healthy controls.^21^ Another study also showed apathy, depression, and motor severity in PD could be predicted by ALFF signals in some brain regions.^22^ Nonetheless, these researchers didn’t consider the possible role of related genetic variations in brain region dysfunction. Hence, in order to identify the gene‐brain‐behavior relationships, we investigated the underlying regulations of BST1 rs4698412 allelic variant in the PD progression by examining ALFF signals along with the correlations between BST1 rs4698412‐modulated brain alterations and clinical symptoms.

## 2 MATERIALS AND METHODS

### 2.1 Participants

According to UK Parkinson’s Disease Society Brain Bank criteria for idiopathic PD,^23^ we recruited 49 right‐handed sporadic PD patients from outpatients and inpatients in the First Affiliated Hospital of Nanjing Medical University. PD patients who were combined with other severe acute or chronic neurological diseases including stroke, brain tumor or psychiatric diseases, were ruled out from our study. Besides, we excluded PD patients with cognitive impairment (Mini‐Mental State Examination (MMSE) score <24). Furthermore, 47 healthy control (HC) subjects were enrolled in our study by advertising in the community. Participants with contraindications for MRI scans or intakes of antidepressant, anxiolytic, or antipsychotic drugs recently were also excluded from this study. Additionally, to minimize conceivable pharmacological impacts on neural activity, PD patients underwent MRI scans and clinical examinations during off‐state (at least 12‐hour withdrawal of pharmacologic treatment for PD). All participants had given their informed consent before the study began, which was approved by the ethics committee of the First Affiliated Hospital of Nanjing Medical University.

### 2.2 Clinical evaluations

All PD patients were assessed their cognitive condition via MMSE^24^ and Frontal Assessment Battery (FAB).^25^ Emotional states were evaluated by means of 17‐item Hamilton Depression Rating Scale (HDRS‐17),^26^ Hamilton Anxiety Rating Scale (HAMA)^27^ and Apathy scale (AS).^28^ Moreover, we employed the motor component of Unified Parkinson’s Disease Rating Scale (UPDRS‐III) and Hoehn and Yahr staging scale (H&Y)^29^ to detect disease severity. Especially, we count tremor scores, rigidity scores, akinesia scores, and postural instability and gait difficulty (PIGD) scores from UPDRS‐III to further evaluate PD progression. Tremor score of each patient was the sum of UPDRS‐III items 20 and 21; rigidity score was the UPDRS‐III items 22; akinesia score was the sum of UPDRS‐III items 23‐26 and 31; PIGD score was the sum of UPDRS‐III items 27‐30. Gait and balance were quantified with Timed Up and Go (TUG) test.^30^ Besides, we assessed the Epworth Sleeping Scale (ESS)^31^ and Fatigue Severity Scale (FSS)^32^ for each PD patient. We also calculated total levodopa equivalent daily dose (LEDD), LEDD of levodopa preparations and LEDD of dopamine receptor agonists in each PD patient of two subgroups, respectively.^33^

### 2.3 DNA isolation and SNP genotyping

Peripheral venous blood samples were obtained from 49 PD patients and 47 HC subjects for genotyping BST1 gene variants. Each subject’s genomic DNA was extracted from 250‐μL EDTA anticoagulant blood using a DNA direct kit (BioTeKe Corporation, Beijing, China) according to the manufacturer’s instructions. The BST1 rs4698412 data were processed and analyzed using MassARRAY TYPER 4.0 software (Agena Bioscience, San Diego, CA, USA) via the Beijing Genomics Institute (BGI). Specially, a polymerase chain reaction (PCR) and single base extension primer were designed by AssayDesigner3.1 software (Agena Bioscience). Each PCR reaction was performed strictly according to the manufacturer’s instructions. After that, the PCR product was disposed of by shrimp alkaline phosphatase (SAP) to remove the dissociative dNTPs that were out of the system. The SpectroCHIP chip after dotting in MassARRAY Nanodispenser RS1000 array machine system (Agena Bioscience) was analyzed by matrix‐assisted laser desorption/ionization time‐of‐flight (MALDI‐TOF) mass spectrometer (Agena Bioscience) and followed by TYPER4.0 software to get the raw data and the genetic diagram.

Afterward, the subjects were classified into different subgroups (A‐allelic variant carriers and GG homozygote carriers) based on previous studies.^7^, ^13^, ^14^, ^34^

### 2.4 MRI acquisition

Participants were scanned via a 3.0 T Siemens MAGNETOM Verio whole‐body MRI system (Siemens Medical Solutions, Erlangen, Germany). Tight foam padding, along with ear‐plugs, was used to minimize head movement and diminish noise. In addition, participants were instructed to hold motionless as much as possible, close their eyes but remain awake, and attempt not to think about anything particularly during the whole scanning procedure. Prior to functional running, three‐dimensional T1‐weighted anatomical images were obtained using the following volumetric 3D magnetization‐prepared rapid gradient‐echo (MP‐RAGE) sequence (repetition time (TR) = 1900 ms, echo time (TE) = 2.95 ms, flip angle (FA) = 9°, slice thickness = 1 mm, slices = 160, field of view (FOV) = 230 × 230 mm^2^, matrix size = 256 × 256). After that, resting‐state functional images were acquired using an echo‐planar imaging (EPI) sequence with the following parameters: TR = 2000 ms, TE = 21 ms, FA = 90°, FOV = 256 × 256 mm^2^, in‐plane matrix = 64 × 64, slices = 35, slice thickness = 3 mm, no slice gap, total volumes = 240 on each participant.

### 2.5 Preprocessing of fMRI data

The images were preprocessed and analyzed via DPARSF software (http://www.restfmri.net/forum/dparsf).^35^ First 10 time points were abandoned. The remaining 230 volumes were kept for slice timing correction and head motion correction. None of the participants with head motions exceeded 2 mm or 2° of translation or rotation in any direction was excluded in our study. We also calculated the mean head translation, mean head rotation, and frame‐wise displacement (FD).^36^ Analysis of these head motion parameters did not reveal any difference between the four subgroups (*P* > 0.05). Afterward, high‐resolution T1 structural images were coregistrated to functional images via a nonlinear image registration approach and segmented using a new segment algorithm with diffeomorphic anatomical registration through exponentiated lie algebra (DARTEL), followed by a 24 parameter Volterra expansion. Finally, fMRI images were spatially normalized into the Montreal Neurological Institute (MNI) template, resampled into a spatial resolution of 3 × 3 × 3 mm^3^ and spatially smoothened with a 4 mm full width at half‐maximum Gaussian kernel.

### 2.6 ALFF processing

The procedure of ALFF calculation was performed using the REST software (http://restfmri.net/forum/REST). In brief, as introduced previously,^37^ all voxels were converted from the time domain to the frequency domain via Fast Fourier Transform (FFT) after image preprocessing. Subsequently, the square root of the power spectrum across 0.01 Hz to 0.08 Hz for each voxel was calculated. The average square root was regarded as ALFF, which was then converted into *z*‐scores for standardization purposes. Afterward, band‐pass filtering (0.01 < *f* < 0.08 Hz) was performed and linear trend was removed.

### 2.7 Statistical Analysis

Demographic and clinical data analysis was performed via IBM SPSS statistics v20.0.0 software (SPSS, Chicago, IL, USA). The comparisons were conducted between PD and HC groups, within BST1 rs4698412 GG carriers and GA/AA carriers. A Kolmogorov‐Smirnov (K–S) test was employed to test for normality. Two‐sample t test or one‐way analysis of variance (ANOVA) was used for normally distributed data. Asymmetrically distributed variables were tested with Mann–Whitney U or Kruskal‐Wallis test. Besides, chi‐square test was for gender. The Hardy‐Weinberg Equilibrium (HWE) of the genotype frequencies were also tested via Chi‐square test. A significant threshold was set at *P* < 0.05.

Two‐way factorial analysis of covariance (ANCOVA: groups × genotypes; groups: PD and HC, genotypes: GG carriers and GA/AA carriers) was performed adjusting for age, gender, and education years, followed by post hoc tests to further explore the main effects and interactions. All statistical thresholds were set at a corrected *P* < 0.001, determined by Monte Carlo simulation for multiple comparisons (http://afni.nimh.nih.gov/pub/dist/doc/manual/AlphaSim.pdf). In addition, Spearman’s correlative analyses with significant thresholds set at *P* < 0.05 were performed between clinical test scores and ALFF values of the clusters showing significant interactions between groups and genotypes.

## 3 RESULTS

### 3.1 Demographic and clinical data

Table 1 demonstrated the genotype frequencies for BST1 rs4698412, which did not deviate from HWE in either group (PD group: *χ* ^2^ = 0.698, *P = *0.403; HC group: *χ* ^2^ = 0.084, *P = *0.772). Demographic characteristics and clinical evaluations of participants among four subgroups were shown in Table 2. No significant effect of diagnosis, genotype, or interaction between diagnosis and genotype was observed for gender and education years. However, age of PD patients was significantly different from HC subjects, which was then taken as a covariant during the following analysis. Additionally, post hoc analysis revealed that no significant difference was found in age between PD‐GG and PD‐GA/AA subgroups (*P* = 0.713). There were no significant differences in MMSE, FAB, ESS, FSS, HDRS‐17, HAMA, and AS scores between GG carriers and GA/AA carriers in PD group. Similarly, H&Y stages, total LEDD, LEDD of levodopa preparations and LEDD of dopamine receptor agonists also matched well between two subgroups in PD. Nevertheless, UPDRS‐III scores (*P* = 0.021) and TUG test time (*P* = 0.001) in GG carriers were significantly different from GA/AA carriers in PD group. Furthermore, there was a significant difference in PIGD scores between PD‐GG and PD‐GA/AA subgroup (*P* = 0.009). But tremor scores, rigidity scores, and akinesia scores in PD‐GG subgroup were not significantly different from PD‐GA/AA subgroup.

**Table 1:** Genotype frequencies for BST1 rs4698412 in PD and HC groups

Genotype	PD (frequency)	HC (frequency)	Total (frequency)
GG	18 (36.7%)	20 (42.6%)	38 (39.6%)
GA	21 (42.9%)	22 (46.8%)	43 (44.8%)
AA	10 (20.4%)	5 (10.6%)	15 (15.6%)

Genotype frequencies for BST1 rs4698412 did not deviate from HWE in either group (PD group: *χ* ^2^ = 0.698, *P = *0.403; HC group: *χ* ^2^ = 0.084, *P = *0.772).

BST1, bone marrow stromal cell antigen‐1; PD, Parkinson’s disease; HC, Healthy control.

**Table 2:** Demographic and clinical evaluation data between PD and HC groups

Items	PD		HC		*p*
	GG carriers (n = 18)	GA/AA carriers (n = 31)	GG carriers (n = 20)	GA/AA carriers (n = 27)	
Ages(y)	67.0 ± 11.4	67.8 ± 6.55	62.2 ± 4.86	63.5 ± 5.11	0.019^a^
Gender(F/M)	4/14	11/20	5/15	13/14	0.237^e^
Education(y)	11.9 ± 3.59	11.2 ± 3.17	12.0 ± 4.48	11.3 ± 2.37	0.632^d^
Disease duration(y)	5.24 ± 4.99	4.69 ± 2.73	NA	NA	0.640^c^
UPDRS‐III score	20.9 ± 9.36	25.9 ± 7.92	NA	NA	0.021^c^^,^ *
Tremor score	4.39 ± 3.33	5.81 ± 3.14	NA	NA	0.142^b^
Rigidity score	5.33 ± 3.41	5.68 ± 2.85	NA	NA	0.459^c^
Akinesia score	6.89 ± 5.22	6.74 ± 3.89	NA	NA	0.676^c^
PIGD score	4.33 ± 2.28	7.65 ± 4.26	NA	NA	0.009^c^^,^ *
H‐Y stage	2.06 ± 0.64	2.16 ± 0.45	NA	NA	0.556^c^
LEDD, mg/day	526 ± 278	453 ± 215	NA	NA	0.304^b^
Dopamine receptor agonists, mg/day	55.6 ± 44.0	46.3 ± 41.3	NA	NA	0.351^c^
Levodopa preparations, mg/day	343 ± 198	281 ± 199	NA	NA	0.295^b^
ESS	4.72 ± 3.56	5.97 ± 4.25	NA	NA	0.303^b^
FSS	29.9 ± 15.3	33.1 ± 15.6	NA	NA	0.536^c^
TUG(s)	12.3 ± 5.21	18.8 ± 6.05	NA	NA	0.001^c^^,^ *
Cognitive performance					
MMSE	28.2 ± 1.38	28.3 ± 1.55	NA	NA	0.605^c^
FAB	15.7 ± 1.45	15.5 ± 2.25	NA	NA	0.832^c^
Mood performance					
HDRS‐17	6.33 ± 4.41	5.90 ± 4.43	NA	NA	0.743^b^
HAMA	10.6 ± 6.49	8.73 ± 4.88	NA	NA	0.275^b^
AS	16.9 ± 8.94	16.7 ± 8.98	NA	NA	0.935^b^

PD, Parkinson’s disease; HC, healthy control; F, female; M, male; UPDRS, Unified Parkinson’s Disease Rating Scale; PIGD, postural instability and gait difficulty; H‐Y, Hoehn and Yahr stages; LEDD, levodopa equivalent daily dose; ESS, the Epworth Sleeping Scale; FSS, Fatigue Severity Scale; TUG, Timed Up and Go; MMSE, Mini‐Mental State Exam; FAB, Frontal Assessment Battery; HDRS, Hamilton Depression Rating Scale; HAMA, Hamilton Anxiety Rating Scale; AS, Apathy Scale. NA, not applicable.

Values were expressed as mean ± *SD*.

Tremor score of each patient was the sum of UPDRS‐III items 20 and 21; rigidity score was the UPDRS‐III items 22; akinesia score was the sum of UPDRS‐III items 23‐26 and 31; PIGD score was the sum of UPDRS‐III items 27‐30.

a One‐way analysis of variance.

b Two‐sample *t* test.

c Mann–Whitney *U* test.

d Kruskal–Wallis test.

e Chi‐square test.

* *P* < 0.05 was considered significant.

### 3.2 ALFF analysis

**Figure 1:**
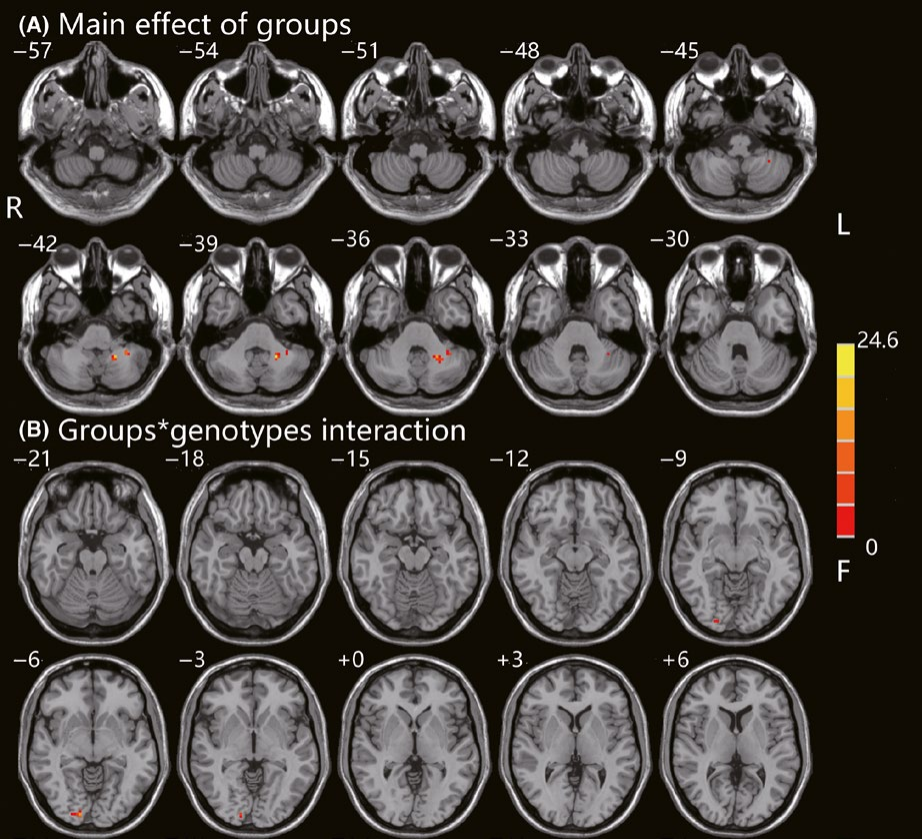
Groups × genotypes ANCOVA of ALFF. (A) Main effect of diagnostic groups on ALFF in PD and HC was shown in the left cerebellum_8 and cerebellum_9. (B) Interaction between BST1 rs4698412 alleles and diagnostic groups was found in the right lingual gyrus. These findings were obtained via two‐way factorial analysis of covariance (ANCOVA: groups × genotypes; groups: PD and HC, genotypes: GG carriers and GA/AA carriers) adjusting for age, gender, and education years. Thresholds were set at a corrected *P < *0.001, determined by Monte Carlo simulation. The color bar indicates the F values from ANCOVA. ALFF, amplitude of low‐frequency fluctuations; PD, Parkinson’s disease; HC, healthy control; BST1, bone marrow stromal cell antigen‐1; R, right; L, left

**Figure 2:**
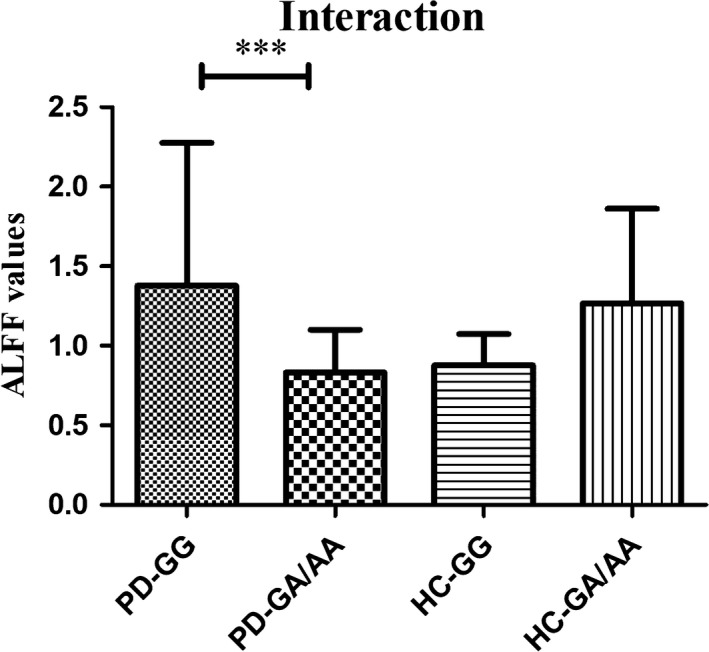
The interaction of groups (PD or HC) and genotypes (GG or GA/AA) on ALFF values. ***Post hoc tests were corrected by Bonferroni correction with a significant different *P* < 0.008 (0.05/6 [number of pair‐comparisons]). The ALFF values were decreased in the right lingual gyrus in PD patients with GA/AA carriers compared to GG carriers and increased in HC subjects. ALFF, amplitude of low‐frequency fluctuations; PD, Parkinson’s disease; HC, healthy control; BST1, bone marrow stromal cell antigen‐1

The influence of diagnosis and genotypes on ALFF between GG and GA/AA carriers in PD and HC groups was shown in Table 3 and Figure 1. Two‐way ANCOVA manifested that the significant main group effect (PD, HC) was observed in left cerebellum_8 (*F* = 16.9, *P* < 0.001, corrected) and cerebellum_9 (*F* = 23.4, *P* < 0.001, corrected). However, no significant different clusters were seen in the main effect of genotypes (GG, GA/AA carriers). Furthermore, interactions between groups and genotypes were found in right lingual gyrus (*F* = 18.0, *P* < 0.001, corrected). Afterward, post hoc tests were used to explore some statistical differences, corrected by Bonferroni correction with a significant different *P* < 0.008 (0.05/6 [number of pair‐comparisons]). Specifically, interaction analysis revealed that GA/AA carriers had decreased ALFF values in right lingual gyrus than GG carriers in PD group (*P* = 0.001), whereas ALFF values in right lingual gyrus were increased in GA/AA carriers compared to GG carriers in HC group (*P* = 0.015). ALFF values in PD‐GA/AA group were a little bit lower than those in HC‐GG group (*P* > 0.05). To some extent, there was a trend that ALFF values in right lingual gyrus were the lowest in PD patients with GA/AA carriers among four subgroups (Figure 2).

**Table 3:** Groups × genotypes ANCOVA of ALFF

Brain region (AAL)	Peak MNI			Peak *F* value	Cluster size (voxels)
	Coordinates x, y, z (mm)				
(1) Main effect of groups					
Cerebellum_8_L	–33	–45	–42	16.9	10
Cerebellum_9_L	–21	–50	–40	23.4	10
(2) Main effect of genotypes					
None					
(3) Groups ×genotypes interaction					
Lingual_R	15	–93	–6	18.0	10

Two‐way factorial analysis of covariance (ANCOVA: groups ×genotypes; groups: PD and HC, genotypes: GG carriers and GA/AA carriers) was performed, adjusting for age, gender, and education years. A corrected threshold by Monte Carlo simulation was set at *P* < 0.001.

PD, Parkinson’s disease; HC, healthy control; ALFF, amplitude of low‐frequency fluctuations; AAL, anatomical automatic labeling; MNI, Montreal Neurological Institute; R, right; L, left.

### 3.3 Correlation analysis

**Figure 3:**
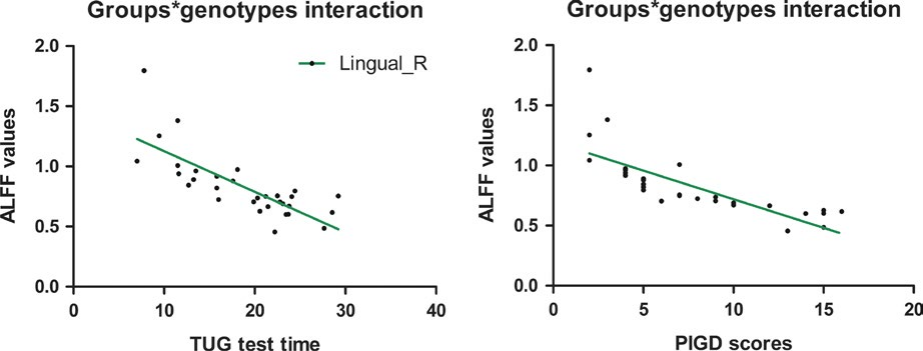
Correlation analysis between TUG test time or PIGD scores and ALFF values in right lingual gyrus. ALFF values in right lingual gyrus affected by interactions between groups and genotypes, were negatively associated with TUG test time (*r* = −0.797, *P* < 0.001) and PIGD scores (*r* = −0.937, *P* < 0.001) in PD individuals carrying GA/AA; ALFF, amplitude of low‐frequency fluctuations; TUG, Timed Up and Go; PIGD, postural instability and gait difficulty; PD, Parkinson’s disease; Lingual_R, right lingual gyrus

ALFF values in right lingual gyrus affected by interactions between groups and genotypes, were negatively associated with TUG test time (*r* = −0.797, *P* < 0.001) and PIGD scores (*r* = −0.937, *P* < 0.001) in PD‐GA/AA group (Figure 3), while no significant association with TUG test time or PIGD scores was observed in the other three subgroups. Furthermore, PIGD scores were positively related to TUG test time (*r* = 0.652, *P* < 0.001). There was no significant correlation between ALFF values in right lingual gyrus and MMSE, FAB, ESS, FSS, HDRS‐17, HAMA, and AS scores (*P* > 0.05). Especially, ALFF values in right lingual gyrus did not significantly correlated to UPDRS‐III scores (*P* = 0.791) and ages (*P* = 0.452) as well. Additionally, there was no correlation between TUG test time and UPDRS‐III scores (*P* = 0.138). Besides, tremor scores, rigidity scores, and akinesia scores did not correlate with TUG test time or ALFF values in right lingual gyrus either (*P* > 0.05).

## 4 DISCUSSION

To our knowledge, this study was the first to investigate the potential effect of BST1 rs4698412 allelic variant on modulating resting‐state brain function in sporadic PD.

BST1 rs4698412 was considered as a candidate SNP for risk of PD supported by several GWAS^7^, ^14^ and a meta‐analysis,^13^ but some other studies also indicated that there was no relationship between BST1 rs4698412 and PD.^34^, ^38^ We hypothesized that the discrepancy might be attributed to heterogeneity of individuals from different populations or experimental methods among studies. To some extent, BST1 rs4698412 might just be a minor gene locus associated with the happening of sporadic PD. But this does not mean any individual carrying the allele will develop PD ultimately. The emergence or development of sporadic PD needs many triggers or accelerating factors including complex interactions among additional genetic variants besides BST1 rs4698412, or interactions between genetic variants and certain environmental exposures.^6^ Besides, plenty of effective treatments can be used to improve clinical symptoms or slow down the progression in PD patients till now. Thus, it is more meaningful to explore the association between BST1 rs4698412 allele and imaging phenotypes (eg ALFF value changes reflecting neural activation in some brain regions) or clinical symptoms than between BST1 rs4698412 allele and susceptibility to sporadic PD.

Our analysis in main effect of genotypes showed that BST1 rs4698412 GA/AA carriers did not significantly differ from GG carriers in resting‐state brain function regardless of disease status. Nonetheless, there was an interaction between diagnostic groups (PD, HC) and genotypes (GG, GA/AA) in right lingual gyrus, which could manifest the differential effects of BST1 rs4698412 genotypes on brain functions in PD and HC. Therefore, both genetic factors and physical conditions should be considered to clarify the potential pathogenic role of BST1 rs4698412 allele. Our study showed that GA/AA carriers had lower ALFF values in right lingual gyrus than GG carriers in PD group, whereas there was a contrary trend between GA/AA carriers and GG carriers in HC group. But the ALFF values did not differ significantly between two subgroups in HC. This further demonstrated that allele A of BST1 rs4698412 contributed to right lingual gyrus deficits during the PD progression. BST1 rs4698412 allelic variant might act as a regulatory role in BST1 gene or in another gene associated with PD, or by other unknown mechanisms to influence the function of lingual gyrus in PD patients. The exact mechanism underlying will be explored in future studies.

Lingual gyrus, located in occipital cortex (also known as visual association cortex), participated in spatial orientation^39^ and visuospatial information processing.^40^, ^41^ Moreover, changes in the flow of visual information mainly dominated by right hemisphere,^42^, ^43^ had an influence on balance disorders^44^ and turning^45^ in PD. Hence, dysfunction in lingual gyrus could cause some problems on walking or balance in PD patients, inconsistent with some previous researches.^46^, ^47^ Interestingly, our study further showed there was a trend that PD‐GA/AA group had the lowest ALFF values in right lingual gyrus among four subgroups and decreased ALFF values were negatively correlated with TUG test time or PIGD scores in this subgroup. Additionally, there was no relationship between ALFF values in right lingual gyrus and TUG test time or PIGD scores in the PD‐GG group. TUG test can be used to evaluate the walking or turning characteristics^48^ and predict fall risks in PD.^49^, ^50^ Besides, PIGD scores from UPDRS‐III scores can also reflect gait or balance function in PD patients to some extent. Taken together, these results could help to interpret the impact of allele A of BST1 rs4698412 on a pathological process leading to gait or balance difficulties in PD patients.

Nevertheless, some limitations should be considered when interpreting our results. Firstly, the sample size of our study was relatively small, so GA carriers and AA carriers were merged into one group, which failed us to explore the respective effects of GA and AA allele on brain function. A larger sample size is necessary based on these preliminary findings in the future. Secondly, only BST1 rs4698412 was investigated in our study while brain spontaneous activities might be affected by various genes and SNPs. Therefore, gene‐gene interactions or more complex haplotype analysis should be further investigated. Thirdly, in this study, we only chose TUG test time to assess gait or balance performance in PD patients. To be rigorous, we should adopt some more tests to further verify our conclusion in future studies. Finally, our present study was a cross‐sectional design focusing on regional brain activation and could not show the causal inference, which needed to be explored in the longitudinal research with multimodal techniques deeply.

## 5 CONCLUSION

The present study demonstrated that functional alternations in right lingual gyrus caused by BST1 rs4698412 allelic variant (allele A) could be associated with gait or balance deficits in PD patients. It might provide a new perspective for further studies on PD for more effective disease intervention. Furthermore, imaging genetics as a new promising approach has the potential to explore relevant neurobiological mechanisms underlying gene polymorphisms on the complicated clinical symptoms in PD.

## CONFLICT OF INTEREST

The authors declare no conflict of interest.

## References

[1] Greenamyre JT , Hastings TG . Biomedicine: Parkinson’s—divergent causes, convergent mechanisms. Science. 2004;304:1120‐1122. 15155938 10.1126/science.1098966

[2] Kalia LV , Lang AE . Parkinson’s disease. Lancet. 2015;386:896‐912. 25904081 10.1016/S0140-6736(14)61393-3

[3] Przedborski S . The two‐century journey of Parkinson disease research. Nat Rev Neurosci. 2017;18:251‐259. 28303016 10.1038/nrn.2017.25

[4] de Lau L , Breteler M . Epidemiology of Parkinson’s disease. Lancet Neurol. 2006;5:525‐535. 16713924 10.1016/S1474-4422(06)70471-9

[5] Qin XY , Zhang SP , Cao C , et al. Aberrations in peripheral inflammatory cytokine levels in Parkinson disease: a systematic review and meta‐analysis. JAMA Neurol. 2016;73:1316‐1324. 27668667 10.1001/jamaneurol.2016.2742

[6] Cannon JR , Greenamyre JT . Gene‐environment interactions in Parkinson’s disease: specific evidence in humans and mammalian models. Neurobiol Dis. 2013;57:38‐46. 22776331 10.1016/j.nbd.2012.06.025 PMC3815566

[7] Satake W , Nakabayashi Y , Mizuta I , et al. Genome‐wide association study identifies common variants at four loci as genetic risk factors for Parkinson’s disease. Nat Genet. 2009;41:1303‐1307. 19915576 10.1038/ng.485

[8] Malavasi F , Deaglio S , Ferrero E , et al. CD38 and CD157 as receptors of the immune system: a bridge between innate and adaptive immunity. Mol Med. 2006;12:334‐341. 17380201 10.2119/2006-00094.Malavasi PMC1829205

[9] Yamamoto‐Katayama S , Ariyoshi M , Ishihara K , et al. Crystallographic studies on human BST‐1/CD157 with ADP‐ribosyl cyclase and NAD glycohydrolase activities. J Mol Biol. 2002;316:711‐723. 11866528 10.1006/jmbi.2001.5386

[10] Lee HC . Physiological functions of cyclic ADP‐ribose and NAADP as calcium messengers. Annu Rev Pharmacol Toxicol. 2001;41:317‐345. 11264460 10.1146/annurev.pharmtox.41.1.317

[11] Chan CS , Gertler TS , Surmeier DJ . Calcium homeostasis, selective vulnerability and Parkinson’s disease. Trends Neurosci. 2009;32:249‐256. 19307031 10.1016/j.tins.2009.01.006 PMC4831702

[12] Michel PP , Hirsch EC , Hunot S . Understanding dopaminergic cell death pathways in Parkinson Disease. Neuron. 2016;90:675‐691. 27196972 10.1016/j.neuron.2016.03.038

[13] Wang S , Xu YF , Ding XY , et al. Association between bone marrow stromal cell antigen 1 gene polymorphisms and the susceptibility to Parkinson’s disease: a meta‐analysis. Neurosci Lett. 2015;599:120‐124. 25986899 10.1016/j.neulet.2015.05.026

[14] Saad M , Lesage S , Saint‐Pierre A , et al. Genome‐wide association study confirms BST1 and suggests a locus on 12q24 as the risk loci for Parkinson’s disease in the European population. Hum Mol Genet. 2011;20:615‐627. 21084426 10.1093/hmg/ddq497

[15] Guo JF , Li K , Yu RL , et al. Polygenic determinants of Parkinson’s disease in a Chinese population. Neurobiol Aging. 2015;36: 1765 e1761‐1765 e1766. 10.1016/j.neurobiolaging.2014.12.030 25623333

[16] Bogdan R , Salmeron BJ , Carey CE , et al. Imaging genetics and genomics in psychiatry: a critical review of progress and potential. Biol Psychiatry. 2017;82:165‐175. 28283186 10.1016/j.biopsych.2016.12.030 PMC5505787

[17] Apostolova LG , Risacher SL , Duran T , et al. Associations of the Top 20 alzheimer disease risk variants with brain amyloidosis. JAMA Neurol. 2018;75:328‐341. 29340569 10.1001/jamaneurol.2017.4198 PMC5885860

[18] Shang CY , Lin HY , Tseng WY , et al. A haplotype of the dopamine transporter gene modulates regional homogeneity, gray matter volume, and visual memory in children with attention‐deficit/hyperactivity disorder. Psychol Med. 2018;48:2530‐2540. 29433615 10.1017/S0033291718000144

[19] Fox MD , Raichle ME . Spontaneous fluctuations in brain activity observed with functional magnetic resonance imaging. Nat Rev Neurosci. 2007;8:700‐711. 17704812 10.1038/nrn2201

[20] Yang H , Long XY , Yang Y , et al. Amplitude of low frequency fluctuation within visual areas revealed by resting‐state functional MRI. NeuroImage. 2007;36:144‐152. 17434757 10.1016/j.neuroimage.2007.01.054

[21] Skidmore FM , Yang M , Baxter L , et al. Reliability analysis of the resting state can sensitively and specifically identify the presence of Parkinson disease. NeuroImage. 2013;75:249‐261. 21924367 10.1016/j.neuroimage.2011.06.056

[22] Skidmore FM , Yang M , Baxter L , et al. Apathy, depression, and motor symptoms have distinct and separable resting activity patterns in idiopathic Parkinson disease. NeuroImage. 2013;81:484‐495. 21782030 10.1016/j.neuroimage.2011.07.012

[23] Hughes AJ , Daniel SE , Kilford L , et al. Accuracy of clinical diagnosis of idiopathic Parkinson’s disease: a clinico‐pathological study of 100 cases. J Neurol Neurosurg Psychiatry. 1992;55:181‐184. 1564476 10.1136/jnnp.55.3.181 PMC1014720

[24] Folstein MF , Folstein SE , McHugh PR . "Mini‐mental state": A practical method for grading the cognitive state of patients for the clinician. J psychiat Res. 1975;12:189‐198. 1202204 10.1016/0022-3956(75)90026-6

[25] Dubois B , Slachevsky A , Litvan I , et al. The FAB: a frontal assessment battery at bedside. Neurology. 2000;55:1621‐1626. 11113214 10.1212/wnl.55.11.1621

[26] Leentjens A , Verhey F , Lousberg R , et al. The validity of the Hamilton and Montgomery‐Åsberg depression rating scales as screening and diagnostic tools for depression in Parkinson’s disease. Int J Geriatric Psychiatr. 2000;15:533‐538. 10.1002/1099-1166(200007)15:7<644::aid-gps167>3.0.co;2-l 10918346

[27] Stefanova E , Ziropadja L , Petrovic M , et al. Screening for anxiety symptoms in Parkinson disease: a cross‐sectional study. J Geriatr Psychiatry Neurol. 2013;26:34‐40. 23407399 10.1177/0891988713476368

[28] Leentjens AF , Dujardin K , Marsh L , et al. Apathy and anhedonia rating scales in Parkinson’s disease: critique and recommendations. Mov Disord. 2008;23:2004‐2014. 18709683 10.1002/mds.22229

[29] Goetz CG , Poewe W , Rascol O , et al. Movement Disorder Society Task Force report on the Hoehn and Yahr staging scale: status and recommendations. Mov Disord. 2004;19:1020‐1028. 15372591 10.1002/mds.20213

[30] Morris S , Morris ME , Iansek R . Reliability of measurements obtained with the Timed "Up & Go" test in people with Parkinson disease. Phys Ther. 2001;81:810‐818. 11175678 10.1093/ptj/81.2.810

[31] Johns MW . A new method for measuring daytime sleepiness the Epworthsleepiness scale. Sleep. 1991;14:540‐545. 1798888 10.1093/sleep/14.6.540

[32] Friedman JH , Alves G , Hagell P , et al. Fatigue rating scales critique and recommendations by the Movement Disorders Society task force on rating scales for Parkinson’s disease. Mov Disord. 2010;25:805‐822. 20461797 10.1002/mds.22989

[33] Tomlinson CL , Stowe R , Patel S , et al. Systematic review of levodopa dose equivalency reporting in Parkinson’s disease. Mov Disord. 2010;25:2649‐2653. 21069833 10.1002/mds.23429

[34] Chang XL , Mao XY , Li HH , et al. Association of GWAS loci with PD in China. Am J Med Genet B Neuropsychiatr Genet. 2011;156B:334‐339. 21268244 10.1002/ajmg.b.31167

[35] Chao‐Gan Y , Yu‐Feng Z . DPARSF: A MATLAB toolbox for "pipeline" data analysis of resting‐state fMRI. Front Syst Neurosci. 2010;4:13. 20577591 10.3389/fnsys.2010.00013 PMC2889691

[36] Power JD , Barnes KA , Snyder AZ , et al. Spurious but systematic correlations in functional connectivity MRI networks arise from subject motion. NeuroImage. 2012;59:2142‐2154. 22019881 10.1016/j.neuroimage.2011.10.018 PMC3254728

[37] Zang YF , He Y , Zhu CZ , et al. Altered baseline brain activity in children with ADHD revealed by resting‐state functional MRI. Brain Dev. 2007;29:83‐91. 16919409 10.1016/j.braindev.2006.07.002

[38] Tan E‐K , Kwok H‐K , Tan LC , et al. Analysis of GWAS‐linked loci in Parkinson disease reaffirms PARK16 as a susceptibility locus. Neurology. 2010;75:508‐512. 20697102 10.1212/WNL.0b013e3181eccfcd PMC2918477

[39] Schiltz C , Bodart JM , Dubois S , et al. Neuronal mechanisms of perceptual learning: changes in human brain activity with training in orientation discrimination. NeuroImage. 1999;9:46‐62. 9918727 10.1006/nimg.1998.0394

[40] Renier LA , Anurova I , De Volder AG , et al. Preserved functional specialization for spatial processing in the middle occipital gyrus of the early blind. Neuron. 2010;68:138‐148. 20920797 10.1016/j.neuron.2010.09.021 PMC2951740

[41] Collignon O , Vandewalle G , Voss P , et al. Functional specialization for auditory‐spatial processing in the occipital cortex of congenitally blind humans. Proc Natl Acad Sci U S A. 2011;108:4435‐4440. 21368198 10.1073/pnas.1013928108 PMC3060256

[42] Jong BMd , Frackowiak R , Willemsen A , et al. The distribution of cerebral activity related to visuomotor coordination indicating perceptual and executional specialization. Cognitive Brain Res. 1999;8:45‐59. 10.1016/s0926-6410(99)00005-1 10216273

[43] Woolley DG , Wenderoth N , Heuninckx S , et al. Visual guidance modulates hemispheric asymmetries during an interlimb coordination task. NeuroImage. 2010;50:1566‐1577. 20079443 10.1016/j.neuroimage.2010.01.012

[44] Suarez H , Geisinger D , Suarez A , et al. Postural control and sensory perception in patients with Parkinson’s disease. Acta Otolaryngol. 2009;129:354‐360. 19021071 10.1080/00016480802495446

[45] Davidsdottir S , Wagenaar R , Young D , et al. Impact of optic flow perception and egocentric coordinates on veering in Parkinson’s disease. Brain. 2008;131:2882‐2893. 18957454 10.1093/brain/awn237 PMC2577802

[46] Cremers J , D’Ostilio K , Stamatakis J , et al. Brain activation pattern related to gait disturbances in Parkinson’s disease. Mov Disord. 2012;27:1498‐1505. 23008169 10.1002/mds.25139

[47] van der Hoorn A , Renken RJ , Leenders KL , et al. Parkinson‐related changes of activation in visuomotor brain regions during perceived forward self‐motion. PLoS One. 2014;9:e95861. 24755754 10.1371/journal.pone.0095861 PMC3995937

[48] Son M , Youm C , Cheon S , et al. Evaluation of the turning characteristics according to the severity of Parkinson disease during the timed up and go test. Aging Clin Exp Res. 2017;29:1191‐1199. 28220396 10.1007/s40520-016-0719-y

[49] Nocera JR , Stegemoller EL , Malaty IA , et al. Using the Timed Up & Go test in a clinical setting to predict falling in Parkinson’s disease. Arch Phys Med Rehabil. 2013;94:1300‐1305. 23473700 10.1016/j.apmr.2013.02.020 PMC4144326

[50] Foreman KB , Addison O , Kim HS , et al. Testing balance and fall risk in persons with Parkinson disease, an argument for ecologically valid testing. Parkinsonism Relat Disord. 2011;17:166‐171. 21215674 10.1016/j.parkreldis.2010.12.007 PMC3042054

